# Exposure to agricultural pesticide impairs visual lateralization in a larval coral reef fish

**DOI:** 10.1038/s41598-017-09381-0

**Published:** 2017-08-22

**Authors:** Marc Besson, Camille Gache, Frédéric Bertucci, Rohan M. Brooker, Natacha Roux, Hugo Jacob, Cécile Berthe, Valeria Anna Sovrano, Danielle L. Dixson, David Lecchini

**Affiliations:** 1 PSL Research University: EPHE-UPVD-CNRS, USR 3278 CRIOBE, BP 1013, 98729 Papetoai, Moorea French Polynesia; 2 Université Pierre et Marie Curie, UMR CNRS 7232 OOB, 1 Avenue Pierre Fabre, 66650 Banyuls-sur-Mer, France; 3 Laboratoire d’Excellence “CORAIL”, BP 1013, 98729 Papetoai, Moorea French Polynesia; 4 Laboratoire de Morphologie Fonctionnelle et Evolutive, AFFISH Research Center, Institut de Chimie B6c, Université de Liège, Liège, Belgium; 5 School of Marine Science and policy, University of Delaware, 111 Robinson Hall, Newark, DE 19716 USA; 6 International Atomic Energy Agency, Environment Laboratories (IAEA-EL), Principality of Monaco, 98000 Monaco; 7 Center for Mind/Brain Sciences (CIMeC), University of Trento, Piazza Manifattura 1, 38068 Rovereto (TN), Italy

## Abstract

Lateralization, *i.e*. the preferential use of one side of the body, may convey fitness benefits for organisms within rapidly-changing environments, by optimizing separate and parallel processing of different information between the two brain hemispheres. In coral reef-fishes, the movement of larvae from planktonic to reef environments (recruitment) represents a major life-history transition. This transition requires larvae to rapidly identify and respond to sensory cues to select a suitable habitat that facilitates survival and growth. This ‘recruitment’ is critical for population persistence and resilience. In aquarium experiments, larval *Acanthurus triostegus* preferentially used their right-eye to investigate a variety of visual stimuli. Despite this, when held in *in situ* cages with predators, those larvae that previously favored their left-eye exhibited higher survival. These results support the “brain’s right-hemisphere” theory, which predicts that the right-eye (*i.e.* left-hemisphere) is used to categorize stimuli while the left-eye (*i.e.* right-hemisphere) is used to inspect novel items and initiate rapid behavioral-responses. While these experiments confirm that being highly lateralized is ecologically advantageous, exposure to chlorpyrifos, a pesticide often inadvertently added to coral-reef waters, impaired visual-lateralization. This suggests that chemical pollutants could impair the brain function of larval fishes during a critical life-history transition, potentially impacting recruitment success.

## Introduction

Brain asymmetry and the preference to use one side of the body over the other to accomplish actions (termed lateralization), has been identified in a variety of vertebrate and invertebrate species^1–5^. Lateralization processes have been characterized in adult organisms but also in early-life-stages, such as fish larvae^6, 7^, fish juveniles^8, 9^, chicks^10^ (but see refs 11–13 for reviews on embryonic and post-embryonic development of lateralized organs and acquisition of lateralized behaviors in several vertebrate and invertebrate species). Processes of lateralization can therefore be expected along the whole ontogeny of an animal organism. Brain lateralization is thought to increase cognitive abilities, in particular for decision making when facing novel multi-sensory signals, by enabling individuals to cope with divided attention and to partition, and optimize, the parallel processing of different types of information into the two separate brain hemispheres^1, 14–18^. With regards to habitat exploration and response to sensory cues, there are two main theories concerning brain lateralization in animals. The “**brain’s right hemisphere theory**” assumes that the right hemisphere is predominantly used to process information regarding novel items and/or items requiring a rapid behavioral response, while the left hemisphere is used for categorizing stimuli and/or processing information that requires consideration of alternatives^19, 20^. Alternatively, the “**valence theory**” proposes that the right hemisphere is preferentially used to process information regarding negative stimuli, while the left hemisphere is used for the processing of information regarding positive stimuli^21–23^. While empirical evidence supports both theories, previous studies have generally only considered lateralized responses to social sensory stimuli under normal environmental conditions, and only in a few species of amphibians, birds, freshwater fishes, and mammals^10, 21, 24–29^. Therefore, we still lack a comprehensive understanding of the ecological importance of sensory lateralization in marine fishes, as well as in the context of rapidly changing environments.

Among the most rapidly changing environments globally are coral reefs, one of the most biologically diverse, productive, and economically important ecosystems worldwide^30–32^. Over the past three decades, coral reefs have faced increasingly frequent and severe natural (*e.g*., cyclones, *Acanthaster planci* outbreaks) and anthropogenic perturbations (*e.g*., pollution from agricultural runoff, rising sea-water temperatures, overfishing) leading to global declines in habitat quality, biodiversity and associated ecosystem services^31, 33–37^. In the context of these rapidly changing conditions, reef fishes often play an important role in maintaining habitat structure, preserving associated biodiversity, and increasing ecosystem resilience (*e.g*., through the regulation of coral-algal interactions or by maintaining trophic chain sustainability)^38–40^. However, these species are often highly vulnerable to changing conditions, with individual health and population persistence impaired by both localized and global impacts^34^. The majority of reef fishes have a bipartite life cycle, consisting of a pelagic larval stage that facilitates dispersal followed by a largely sedentary reef-associated juvenile and adult stage^41^. At the conclusion of the larval phase, the transition from the planktonic to reef environments is referred to as settlement^41^. During this transition, reef-naïve larvae metamorphose into benthic dwelling juveniles and must rapidly identify and respond appropriately to a host of novel multi-sensory signals in order to select a suitable habitat that will facilitate their survival and growth^42–49^. The selection of, and persistence in, an appropriate habitat is termed recruitment^41^. Continued and consistent recruitment is essential to maintain reef fish populations and assist with the resilience and recovery of degraded reef fish communities^50^. The ability to identify visual cues that indicate the identity and location of predators has shown to increase survival rates during recruitment in a number of coral reef fish species^45, 46, 51–53^.

The relationship between brain morphology, lateralization, social interactions, and anti-predator behaviors in early-life-stage coral reef fishes remains largely unknown^8, 45, 46, 51, 54–56^, with the role of behavioral lateralization during recruitment only initially assessed^55, 56^. These recent experiments have primarily used detour tests, which examine potential turning bias of individuals in a context of vigilance, and revealed that such lateralized behaviors can be associated with anti-predator response phenotypes and survival benefits^55–57^. However, these studies were conducted on captive-bred larvae, larvae caught in light traps (which may selectively attract certain behavioral phenotypes), or wild caught post-settlement stage juveniles. Experimental use of individuals that have been reared, are from mixed larval, pre-settlement, and post-settlement stages, or are non-naïve and have past reef experience potentially adds biases to these findings. This prevents a complete understanding of the ecological importance of coral reef fish lateralization at the critical time of recruitment. In addition, current research has yet to examine the contribution of either brain hemisphere in accomplishing a specific task.

Short-term exposure to environmental stressors, such as high predation risk or elevated CO_2_ levels, has been shown to alter lateralized anti-predator behaviors, which can lead to greater mortality rates^55, 57^. These findings highlight the critical role of lateralization during the recruitment in coral reef fishes, and point towards potential population or ecosystem-level impacts when an environmental stressor is applied^56–59^. In particular, anthropogenic impacts on reefs, such as the worldwide increasing influx of chemical pollution (*e.g*., from pesticides) resulting from coastal agriculture and river runoff^60–69^, are known to severely impact reef biodiversity and the biology and ecology of coastal marine organisms^53, 62^. Among these sources of pollution, the Chlorpyrifos (CPF), an organophosphate insecticide widely used on tropical coastal crops (*e.g*., sugar-cane and rice crops in Australia and south-east Asia respectively^64, 70, 71^), is one of the most common waterborne chemical pollutant encountered in coral reefs^70, 71^. While several studies have demonstrated its neurotoxicity and endocrine disruption characteristics^71–74^, the negative impact on coral reef larval fish sensory abilities has also recently been acknowledged^71^. Moreover, pesticides belonging to the same family have been discovered in important amounts in several fish and crustacean species in French Polynesia^68, 75^. However, whether this pollutions affect or not behavioral lateralized processes remains unknown^71^.

**Figure 1:**
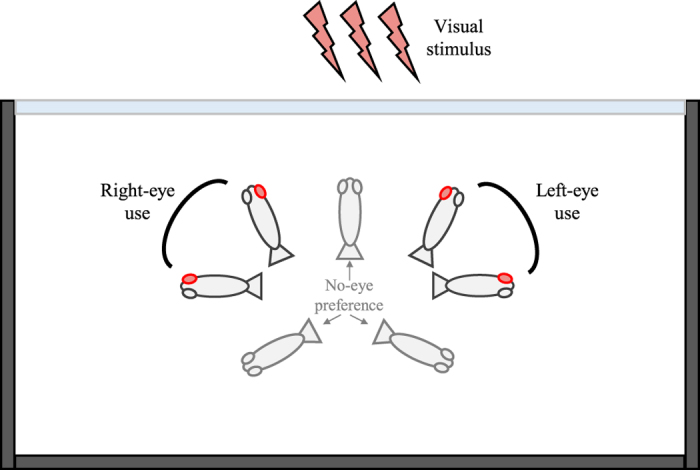
Schematic representation of the test apparatus for eye lateralization determination. The three dark gray walls represent the opaque wall of the aquarium, while the light blue wall corresponds to the transparent wall where the visual stimuli are presented. No eye preference was recorded when the fish was perpendicular to the transparent wall (*i.e*. binocular stimulation) or when it formed an angle larger than 180° with respect to the transparent wall (*i.e*. fish looking in the opposite direction).

Here, we examined visual lateralization in the larvae of a common coral reef fish species, the convict surgeonfish *Acanthurus triostegus* ^76^, during its recruitment phase. If settlement-stage larvae preferentially use their right eye to examine and categorize positive stimuli (*e.g*., conspecifics, such as in *Myrispristis pralinia*, a coral reef fish^8^, or in freshwater fishes^17, 24, 25, 77^) and their left eye for negative stimuli or to inspect novel items and execute rapid responses (*e.g*., potential predators, such as in freshwater fishes^17, 24, 25, 77^), this would be good evidence for both the “**brain’s right hemisphere**” and the “**valence**” theories. Using a range of aquarium based (Fig. 1) and *in situ* experiments we tested whether larvae exhibit a naïve preference for their left or right eye when inspecting both positive (mirror self-image, conspecific) or negative (predator) stimuli (Exp. 1) and whether visual lateralization increased or decreased vulnerability to predation (Exp. 2). Lastly, to examine the potential for coastal development to affect this mechanism we determined whether exposure to CPF, at ecologically relevant concentration^64, 71, 78^, would alter the degree of lateralization larvae exhibit (Exp. 3).

## Results

For each individual within each experiment, a lateralization index (LI), reflecting the degree of preferential eye use, was calculated as: [(frequency of right eye use)/(frequency of right eye use + frequency of left eye use)] × 100. Values significantly higher than 50% indicate preference for right eye use while values significantly lower than 50% indicate preference for left eye use. Subsequent statistical tests were conducted using these values.

**Figure 2:**
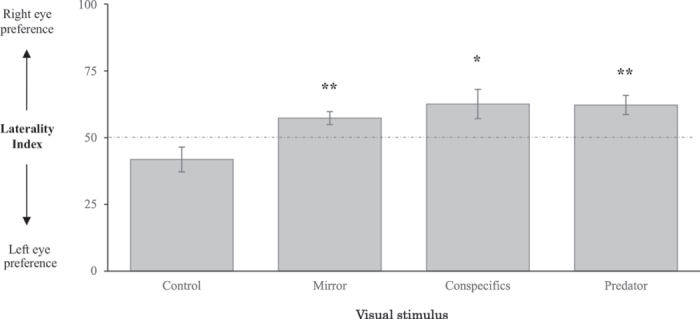
Left-right eye preference of *A. triostegus* larvae during the inspection of visual stimuli in an adjacent aquarium. Figure represents LI mean (±SE) values. Asterisks indicate significant differences (Mann-Whitney U tests, *p < 0.05; **p < 0.01) between Laterality Index (LI) values and the theoretical 50% value (dotted line). 13 to 14 replicates (one fish per replicate) were conducted for each visual stimulus.

**Exp. 1** tested whether larvae exhibit a naïve eye preference when inspecting visual cues from positive (conspecific) or negative (predator) stimuli. In the absence of any visual stimulus, *A. triostegus* larvae exhibited no eye preference (LI = 41.8 ± 4.6%, Mann-Whitney U test, U = 32, n = 14, p-value = 0.364) (Fig. 2). However, larvae showed a significant preference when inspecting test stimuli (Kruskal-Wallis rank sum test, *x* ^2^ = 14.173, df = 3, p-value = 0.003), with LI values significantly higher when examining both conspecifics (Nemenyi post-hoc test with Tukey-Dist approximation, p-value = 0.006) or predator cues (p-value = 0.006). With regards to the eye preferred, larvae displayed a significant preference for their right eye when inspecting themselves in a mirror (LI = 57.3 ± 2.4%, Mann-Whitney U test, U = 93, n = 14, p-value = 0.009), when examining a group of five adult conspecifics (LI = 62.5 ± 5.5%, U = 85, n = 14, p-value = 0.042), or when examining a predator (LI = 62.2 ± 3.6%, U = 83, n = 13, p-value = tested whether visual lateralization increased 0.006) (Fig. 2).

**Figure 3:**
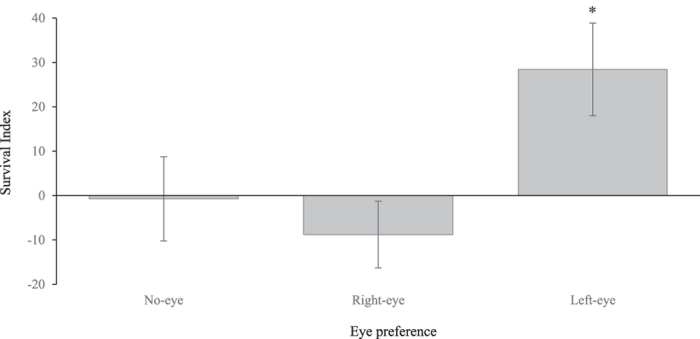
Ecological importance of brain lateralization in larval survival facing direct predation. Survival index (SI) was calculated as follows: survival rate of the group minus the overall survival rate in the *in situ* cage. Figure represents SI mean ( ± SE) values. The asterisk indicates a significant difference in survival index among the three groups (Kruskal-Wallis test, *x* ^2^ = 7.064, df = 2, p-value = 0.029) with ‘left-eye dominant’ experiencing higher survival than both ‘right-eye dominant’ (Nemenyi post-hoc test with Tukey-Dist approximation, p-value = 0.01) and ‘no eye dominant’ larvae (Nemenyi post-hoc test with Tukey-Dist approximation, p-value = 0.03). Six replicates were conducted. In each replicate, each group was made up with the same amount (four to six) of fish.

**Exp. 2** or decreased vulnerability to predation. Individual larvae from Exp. 1 were classified and grouped as either ‘no eye dominant’, ‘right-eye dominant’, or ‘left-eye dominant’ based on the behavior displayed when exposed to the predator in Exp. 1. All three groups experienced significantly different survival rates (Kruskal-Wallis rank sum test, *x* ^2^ = 7.064, df = 2, p-value = 0.029) with ‘left-eye dominant’ larvae having a higher survival rate than both ‘right-eye dominant’ (Nemenyi post-hoc test with Tukey-Dist approximation, p-value = 0.01) and ‘no eye dominant’ larvae (p-value = 0.03) (Fig. 3).

**Figure 4:**
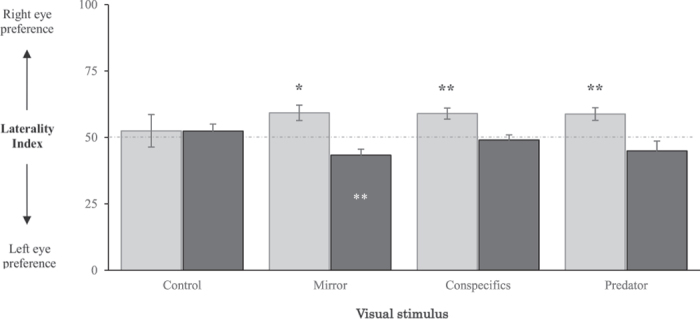
Effect of pesticides on the visual preference of *A. triostegus* larvae. Figure represents LI mean (±SE) values. Asterisks indicate significant differences (Mann-Whitney U tests, *p < 0.05; **p < 0.01) between Laterality Index (LI) values and the theoretical 50% value (dotted line). Black asterisks above bars indicate a significant right-eye preference, while white asterisks below bars indicate a significant left-eye preference. 10 to 11 replicates (one fish per replicate) were conducted for each visual stimulus in both the pesticide exposition experiment (black bars) and the solvent-control exposition experiment (grey bars).

**Exp. 3** utilized the same protocol as in Exp. 1 and tested whether exposure to a waterborne organophosphate pesticide often encountered in coral reefs: chlorpyrifos (CPF), would alter the degree of lateralization larvae exhibit. Larvae exposed to CPF at 1 µg.l^−1^ (black bars in Fig. 4) and larvae only exposed to the CPF solvent (grey bars in Fig. 4) again showed no eye preference in the absence of visual stimuli (respectively LI = 52.5 ± 6.1% and 52.3 ± 2.7%, Mann-Whitney U test with respectively U = 33 and 35, n = 10 and 11, p-value = 0.625 and 0.476) (Fig. 4). However, a preference was seen when these larvae inspected a mirror (Wilcoxon rank sum test, W = 106, n = 10, p-value < 0.001), conspecifics (W = 87, n = 10, p-value = 0.004) or a predator (W = 85, n = 10, p-value = 0.009). Similar to the trends seen in Exp. 1 (Fig. 2), solvent control larvae preferentially used their right eye when inspecting a mirror (LI = 59.2 ± 2.9%, Mann-Whitney U test, U = 61.5, n = 11, p-value = 0.013), conspecifics (LI = 58.9 ± 2.1%, U = 54, n = 10, p-value = 0.004) or a predator (LI = 58.8 ± 2.4%, U = 45, n = 10, p-value = 0.009) (Fig. 4). However, when exposed to CPF at 1 µg.l^−1^ for five days, larvae no longer exhibited any eye preference when inspecting either the conspecifics or predator (respectively LI = 49.0 ± 1.9% and 44.9 ± 3.7%, U = 25 and 16, n = 10 and 10, p-value = 0.846 and 0.262). In addition, when inspecting themselves in a mirror, CPF treated larvae now preferentially used of their left eye (LI = 43.3 ± 2.2%, U = 1, n = 10, p-value = 0.004) (Fig. 4).

## Discussion

This study identified a strong preference for the right eye when inspecting stimuli from either a mirror, conspecifics, or a predator. While a preference for the using the right eye, or the left hemisphere of the brain, to categorize familiar stimuli (*e.g*. same size conspecifics) has also been seen in another coral reef fish: *Myrispristis pralinia* ^8^, our results suggest that this preference also extends to unfamiliar stimuli. Larvae used in this experiment were naïve without any prior experience of non-larval reef fishes, therefore adult conspecifics and predators are considered unfamiliar stimuli while mirror-images are considered as the image of a familiar conspecific (*e.g*. larva from the same larval cohort)^27^. The preference for the right eye is intriguing since it is opposite to the preference for the left eye generally observed during the inspection of mirror self-images in freshwater fishes and amphibians, and which conforms with the key role the right telencephalon hemisphere plays in social cognition^10, 21, 24–29, 77, 79–81^. The difference suggests that patterns of laterality could be associated with habitat preference (*e.g*. freshwater *vs*. sea water) or evolutionary history, and thus driven by ecological pressures or phylogenetic constraints. For instance, the preferential use of the right eye could reflect a greater need for settlement-stage *A. triostegus* to initially categorize new sensory cues upon recruitment rather than establishing social interactions and aggregations^82^.

We also observed that larvae that displayed a left-eye preference when inspecting a predator held in an adjacent aquarium in Exp. 1, experienced higher survival when exposed to predation risk (Exp. 2). This result could be attributed to a learning process, with right-brain lateralized individuals able to recognize previously-experienced threats faster than left-brain lateralized individuals when this has been examined in other fishes^83^. Left-eye dominant larvae could potentially escape predation more efficiently. Even if it is unknown if the right or left eye dominated in Exp. 2 itself, it has been shown that an eye preference displayed during the first observation of a predator (as in Exp. 1) can influence behavioral responses (*e.g*. subsequent eye preference, turning rates) when facing for a second time that same, no longer unknown, stimulus^84, 85^. Therefore, after the Exp. 1 conditioning, the left-eye users may adopt a behavioral response to predation that differs from the majority of their right-eye user and not-lateralized conspecifics and that could favor their survival (*e.g*. by disorienting predators or by moving differently within their environment such as seen in mosquitofish^86^). Overall, our results strongly support the “brain’s right hemisphere” theory that predicts that the right-eye (i.e. left-hemisphere) is predominantly used to categorize stimuli, while the left-eye (i.e. right hemisphere) is dedicated to attending to events that require rapid behavioral responses, such as predation^19, 20^. In the context of larval settlement, where individuals face a variety of novel sensory cues and constantly high predation risk, our study suggests that there is a survival advantage to being lateralized, with the ability to optimize survival overcoming the obvious disadvantages of displaying predictable asymmetric behaviors. Future experiments brain lateralization in coral reef fishes should include juveniles that have been allowed to contact with older conspecifics, or that are no longer predator naïve, in comparison to others held in solitary confinement, to better understand how asymmetric behavioral patterns are adopted by a population and the importance of learning and selection in lateralization processes.

Our results show that the presence of chlorpyrifos (CPF), one of the most common waterborne pesticides encountered in coral reefs^71^, can completely diminish or reverse critical lateralization patterns (Fig. 4). Oceanic levels of chlorpyrifos contamination have only been assessed, to our knowledge, in the Southern Ocean, revealing levels up to 0.54 pg.l^−1^ ^87^. This is much lower than the levels observed in Australian reef surface waters, where contamination have been shown to reach 1 µg.l^−1^ ^78^, or than in certain Australian and Malaysian river mouths, where contamination levels can reach up to 26 µg.l^−1^ ^64, 78^. The concentration of 1 µg.l^−1^ is therefore ecologically relevant and reflects appropriately the change of pesticide concentration larval fishes can face when settling, from the open ocean, in coral reef coastal areas^64, 71, 78, 87^. This loss and reversal of lateralization in fish exposed to chlorpyrifos may further increase larval mortality and impact the subsequent recruitment and maintenance of reef ichthyologic diversity, as evidenced in Exp. 2 (Fig. 3). Similar findings have been documented in the coral reef fish *Pomacentrus wardi* when juveniles were exposed to future ocean acidification conditions^59^. In addition, a turning bias, shifting from the right to the left, was observed in *P. wardi* populations exposed to elevated-CO_2_ concentrations (930 µatm)^58^. Lastly, the same study also revealed how increased temperatures (+3 °C higher than ambient temperatures) can have a dampening effect on lateralization processes^58^. In the context of global degradation of coral reefs, it is striking to observe that pesticide pollution, oceanic acidification, and elevated water temperatures could have similar and potentially interactive effects in impairing lateralized behaviors of marine organisms and subsequent survival to predation, particularly in early developmental stages. With this study, we demonstrate how the worldwide increasing influx of pesticide onto coral reefs, due to expanding coastal agriculture, as well as changing patterns of land use upstream^60, 61, 63–69^, may lead to a loss or reversal of visual lateralization during a critical step of coral reef fish life cycle. This may potentially lead to greater larval mortality rates and reduce subsequent recruitment success. In addition, any potential reduction in the abundance of major herbivorous fishes in coastal marine ecosystems, such as *A. triostegus* in coral reefs^76^, due to chemical pollution from organophosphate components^88^, could impair the ability of reefs to cope with increasing macro-algal abundance and impact ecosystems recovery and resilience^40, 89^.

While coral reefs are experiencing increasing perturbations^34^, persistence of species in degraded areas rely on the ‘rescue’ effect of recruitment^50^. The potential for a population to be supplemented by recruits depends largely on pelagic larvae detecting an appropriate habitat, towards which they orientate in order to settle and persist^50^. Successful recruitment of individuals could be partly jeopardized by the alteration of lateralized cognitive abilities of larvae caused by local stressors such as pesticides. An understanding of the underlying mechanisms of these physiological and behavioural disturbances requires further investigations.

## Material and Methods

### Sampling and study site

Fish larvae were collected nightly from January to June 2016 using a crest net set on the north-east coast of Moorea Island, French Polynesia (17°29′52.19″S, 149°45′13.55″W). This crest net had a mesh size of 1 mm and was equipped with a rectangular mouth (width: 2 m, height: 1 m) oriented perpendicular to the water flow. Crest nets retain all settlement-stage larvae at the site as they move over the reef crest^90–93^. This sampling technique ensures a non-selective capture (as opposed to light-traps) of wild larval fishes, precisely at the settlement stage, with no prior reef experience (as opposed to the capture of post-settlement juveniles within reefs using light traps or nets)^94^. While this method also permits the study of wild individuals caught *in situ* (as opposed to aquarium reared larval fish), it however does not ensure that the oceanic experience was the same for all larvae. As larval fish primarily recruit to reefs at night^41^, the net was set up at 7 p.m. and larvae were collected at 6 a.m. the following day.

The convict surgeonfish, *Acanthurus triostegus (*Linnaeus, 1758) has a pelagic larval duration of 53 ± 8 days, after which larvae metamorphose and recruit in shoals on shallow sandy and rubble reef areas^48, 76, 95^. This species was chosen as our model due to the high number of larvae collected in the crest net (n = 278 larvae, size: 23 ± 2 mm). After removal from the crest net, larvae were transferred to CRIOBE research station where they were maintained in individual aquaria filled with UV-sterilized and filtered (10 µm filter) seawater before being tested. Water temperature was maintained at 28.5 °C, under 12:12 LD cycle (06:00, onset – 18:00, offset). For Exp. 1 and Exp. 2, tests were conducted immediately following collection (within 12 hours). Exp. 3 tests were conducted on the fifth day post-collection. Throughout their entire time in captivity fish were fed a commercial dry food twice daily.

### Exp. 1: Do A. triostegus larvae exhibit a left-right visual preference when inspecting either negative (predator or positive (mirror self image, conspecific) stimuli?

Preferences for using the left or right eye to inspect visual stimuli was assessed using a protocol adapted from studies of brain lateralization in freshwater fishes^24, 25, 27, 77^. The experimental aquarium was a rectangular glass tank (20 L × 11 W × 15 cm H) with all sides covered with white opaque screen except one of the long sides where stimuli were presented. The tank was lighted with a neon lamp (45 W) and filled with UV-sterilized, filtered (10 µm filter) seawater to 15 cm depth. A video camera was mounted above the experimental tank to record the fish position.

Four types of visual stimuli were presented to *A. triostegus* larvae:

(i). **Control**: a white opaque screen was displayed on the transparent wall, so that all walls of the experimental tank were covered with white opaque screens. This control was conducted to account for any effect of the experimental procedure on fish behavior.
(ii). **Mirror**: a mirror was placed along the transparent wall, presenting larvae with a visual stimulus of their own reflection.
(iii). **Conspecific**: an aquarium (30 × 20 × 20 cm) containing juvenile conspecifics (n = 5, size: 80 ± 4 mm) was placed against the transparent wall. The conspecifics were collected with hand nets in shallow sandy areas surrounded by corals at Moorea Island.
(iv). **Predator**: similarly to (iii), a common reef predator of *A. triostegus* (Besson pers. obs.), *Lutjanus fulvus*, (n = 1, size: 162 mm) was presented in an aquarium placed against the transparent wall of the experimental aquarium.

For each stimulus, 14 biological replicates were conducted (one larva per replicate). Between each replicate, all water in the experimental tank was replaced. For each replicate, the use of the right or left monocular visual field (*i.e*. eye use) was assessed every two seconds for a total of 10 min. Discrimination between the use of the right or the left eye was determined by examining the angle between larva’s body orientation and the transparent wall (where the visual stimulus was presented, Fig. 1), following the method established by Sovrano *et al*.^24^ and used in various eye lateralization studies since^17, 25, 27, 77, 79^. If the fish was perpendicular to the transparent wall (*i.e*. binocular stimulation), or if its body orientation to the transparent wall formed an angle larger than 180° with respect to the transparent wall (*i.e*. fish looking in the opposite direction), no eye preference was recorded^25, 79^ (Fig. 1). In this study, binocular stimulation never occurred (in all three experiments). Moreover, when a visual stimulus was presented, the times when an eye use preference could not be established (because of the fish looking in the opposite direction of the stimulus) only represented 24.01 ± 2.25% of the observations in Exp. 1, 23.78 ± 5.82% of the observations in Exp. 2, and 24.20 ± 2.02% in Exp. 3. Therefore, when examining a visual stimulus, fishes spent 75% of the time examining this visual stimulus with either their left or their right eye, and the rest of the time looking in the opposite direction, but never examining the stimulus with both eyes. Observations of the left-eye or right-eye use (Fig. 1) resulted in a lateralization index (LI), reflecting relative eye use: LI = [(frequency of right eye use)/(frequency of right eye use + frequency of left eye use)] × 100 for each stimulus tested^24^. Values significantly higher than 50% indicate a preference for the right eye, whereas values significantly lower than 50% indicate a preference for the left eye. For each stimulus, the difference between the measured LI and a ‘no eye preference’ case (LI = 50%) was tested using a two-tailed Mann-Whitney U test, in order to identify any potential eye preference. Further statistical analyses to compare LI values between each stimulus were carried out by non-parametric analysis of variance (Wilcoxon rank sum test and Kruskall-Wallis test). For all stimuli, no significant difference was observed between the first 5 min of the trial and the last 5 min of the trial (Kruskal-Wallis test, *x* ^2^ = 92.8, df = 91, p-value = 0.42). To verify that our modified protocol (one single transparent window) did not introduce any behavioral bias compared to the more commonly used protocol (two transparent windows equipped with two mirrors), we performed an additional two-mirror stimulus where an aquarium was equipped with two transparent walls and two mirrors^24, 25, 27^. No significant difference was found when comparing LI values obtained in this two-mirror protocol and the values obtained with the use of the one-mirror protocol (respectively LI = 59.8% and 57.3%, Wilcoxon rank sum test, W = 105, n = 14, p-value = 0.769), therefore we assume that our adapted method provides results comparable to conventional methods. All statistical analyses were conducted using the R-Cran software (R-3.3.1).

### Exp. 2: Ecological importance of brain lateralization in larvae facing predation

Using a similar protocol as Exp. 1, larvae were tested individually to determine if they preferentially used the right eye, left eye, or displayed no lateralization when presented with a predator. Larvae were then classified and pooled into three groups according to their visual preference: ‘left-eye dominant’, ‘right-eye dominant’ and ‘no eye dominant’ individuals. Each group was tagged with a different color, using visible implant fluorescent filament (Northwest Marine Technology), at least one hour before being released simultaneously into a caged patch reef (1 × 1 × 1 m), built from rubble and live coral, and located in an open sandy area at 2 m depth. After a one hour habituation period, three predators, *Lutjanus fulvus* (n = 3, size: 165 ± 14 mm), were introduced into the cage. Prior to their introduction, the predators were held for 48 h in aquaria without food to ensure that they were motivated to feed. The experiment was stopped after two hours, predators were removed and the remaining larvae were counted and identified according to their color tag. The overall survival rate was recorded, as well as a survival rate for each group. For each group, a survival index (SI) was calculated: SI = survival rate of the group – overall survival rate in the cage. SI values allowed a comparison between the survivals of each replicate, regardless of the relative predation rate in each technical replicate. This experiment was replicated 6 times, and each time a different tag color was attributed to the groups to account for any potential predation bias towards one color. For each replicate, all larval groups contained the same number of fish (between 4 and 6 larvae per group depending on the replicate). Statistical analyses to compare SI values were conducted by using non-parametric analysis of variance (Wilcoxon rank sum test and Krukall-Wallis test, followed by Nemenyi post-hoc test should a significant difference be detected).

### Exp. 3: Effect of chemical pollution on the visual lateralization of *A. triostegus* larvae

Preliminary experiments were conducted on three groups of n = 10 fish maintained in 30 L × 20 W × 20 cm H aquaria, which were exposed, after collection in the crest net, to chlorpyrifos (CPF) at 1, 30 and 100 µg.l^−1^, as previously done in the literature in another coral reef fish^71^. After 10 days of exposure, larvae at 30 µg.l^−1^ presented an irregular swimming behavior and most larvae at 100 µg.l^−1^ died after only one day of exposure. Larvae exposed to the 1 µg.l^−1^ concentration exhibited no behavioral abnormality and no mortality. Consequently, we tested the effect of a CPF pollution on larvae visual lateralization through two experimental conditions: (A) solvent control (9 liters of UV-sterilized and filtered −10 µm filter – seawater + 9 µl of acetone) and (B) 1 µg.l^−1^ of CPF (9 liters of UV-sterilized and filtered − 10 µm filter – seawater + 9 µg of CPF diluted in 9 µl of acetone).

For each condition, larvae were tested for their eye preference as in Exp. 1, when presented with the same visual stimuli as in Exp. 1 (control, mirror, conspecifics and predator), after a five day exposure (to CPF or to the solvent). Consequently, we performed n = 8 (2*4) experiments (control, mirror, conspecifics and predator visual stimulus with fish exposed to solvent *vs*. fish exposed to CPF at 1 µg.l^−1^). For each of those 8 experiments, *A. triostegus* larvae were maintained, after collection in the crest net, in groups of n = 5 to 6 fish in 30 L × 20 W × 20 cm H aquaria. Each aquarium was equipped with an air stone. All water was replaced every day, ensuring the maintenance of water quality as well as a continuous concentration of the pesticide or solvent. A second technical replicate for these 8 experiments, with significantly similar results as in the first replicate, was performed. Consequently n = 10 to 11 biological replicates were conducted for each of these 8 experiments. Statistical tests for LI values analyses were performed as described in Exp. 1.

### Ethical approval

All the experiments were approved by the CRIOBE-IRCP animal ethics committee and performed in accordance with the guidelines of the French Polynesia committee for publication and animal ethics.

## References

[1] Rosa Salva O, Regolin L, Mascalzoni E, Vallortigara G (2012). Cerebral and Behavioural Asymmetries in Animal Social Recognition. Comp. Cogn. Behav. Rev..

[2] Frasnelli E, Vallortigara G, Rogers LJ (2012). Left-right asymmetries of behaviour and nervous system in invertebrates. Neurosci. Biobehav. Rev..

[3] Vallortigara G, Rogers LJ, Bisazza A (1999). Possible evolutionary origins of cognitive brain lateralization. Brain Res. Rev..

[4] Vallortigara G, Chiandetti C, Sovrano VA (2011). Brain asymmetry (animal). Wiley Interdiscip. Rev. Cogn. Sci..

[5] Bisazza A, J. Rogers L, Vallortigara G (1998). The origins of cerebral asymmetry: A review of evidence of behavioural and brain lateralization in fishes, reptiles and amphibians. Neurosci. Biobehav. Rev..

[6] Dadda M, Domenichini A, Piffer L, Argenton F, Bisazza A (2010). Early differences in epithalamic left-right asymmetry influence lateralization and personality of adult zebrafish. Behav. Brain Res..

[7] Barth KA (2005). fsi zebrafish show concordant reversal of laterality of viscera, neuroanatomy, and a subset of behavioral responses. Curr. Biol..

[8] Roux N (2016). Brain lateralization involved in visual recognition of conspecifics in coral reef fish at recruitment. Anim. Behav..

[9] Roussigné M, Blader P, Wilson SW (2012). Breaking symmetry: The zebrafish as a model for understanding left-right asymmetry in the developing brain. Dev. Neurobiol..

[10] Vallortigara G, Andrew RJ (1991). Lateralization of response by chicks to change in a model partner. Anim. Behav..

[11] Cooke J (2004). Developmental mechanism and evolutionary origin of vertebrate left/right asymmetries. Biol. Rev..

[12] Levin M (2005). Left – right asymmetry in embryonic development: a comprehensive review. Mech. Dev..

[13] Güntürkün O, Ocklenburg S (2017). Ontogenesis of Lateralization. Neuron.

[14] Rogers LJ (2000). Evolution of Hemispheric Specialization: Advantages and Disadvantages. Brain Lang..

[15] Vallortigara G (2000). Comparative neuropsychology of the dual brain: a stroll through animals’ left and right perceptual worlds. Brain Lang..

[16] Rogers LJ, Zucca P, Vallortigara G (2004). Advantages of having a lateralized brain. Proc. Biol. Sci..

[17] Sovrano VA, Dadda M, Bisazza A (2005). Lateralized fish perform better than nonlateralized fish in spatial reorientation tasks. Behav. Brain Res..

[18] Vallortigara, G. & Rogers, L. J. Survivazl with an asymmetrical brain: advantages and disadvantages of cerebral lateralization. *Behav. Brain Sci*. **28**, 575-89–633 (2005). 10.1017/S0140525X05000105 16209828

[19] Rogers, L. J., Vallortigara, G. & Andrew, R. J. *Divided Brains: the Biology and Behaviour of Brain Asymmetries*. *Cambridge University Press* (2013).

[20] MacNeilage PF, Rogers LJ, Vallortigara G (2009). Origins fo the Left & Right Brain. Sci. Am..

[21] Hook-Costigan MA, Rogers LJ (1998). Eye preferences in common marmosets (Callithrix jacchus): influence of age, stimulus, and hand preference. Laterality.

[22] Siniscalchi M, Lusito R, Vallortigara G, Quaranta A (2013). Seeing left- or right-asymmetric tail wagging produces different emotional responses in dogs. Curr. Biol..

[23] Quaranta A, Siniscalchi M, Vallortigara G (2007). Asymmetric tail-wagging responses by dogs to different emotive stimuli. Curr. Biol..

[24] Sovrano VA, Rainoldi C, Bisazza A, Vallortigara G (1999). Roots of brain specializations: Preferential left-eye use during mirror- image inspection in six species of teleost fish. Behav. Brain Res..

[25] Sovrano VA, Bisazza A, Vallortigara G (2001). Lateralization of response to social stimuli in fishes: A comparison between different methods and species. Physiol. Behav..

[26] Sergent, J., Signoret, J.-L., Bruce, V. & Rolls, E. T. Functional and anatomical decomposition of face processing: evidence from prosopagnosia and PET study of normal subjects. *Philos. Trans. R. Soc. Lond. B. Biol. Sci.* **335**, 55-61–2 (1992). 10.1098/rstb.1992.0007 1348138

[27] Sovrano VA (2004). Visual lateralization in response to familiar and unfamiliar stimuli in fish. Behav. Brain Res..

[28] Bisazza A, De Santi A, Bonso S, Sovrano VA (2002). Frogs and toads in front of a mirror: Lateralisation of response to social stimuli in tadpoles of five anuran species. Behav. Brain Res..

[29] Dadda M, Sovrano VA, Bisazza A (2003). Temporal pattern of social aggregation in tadpoles and its influence on the measurements of lateralised responses to social stimuli. Physiol. Behav..

[30] Odum HT, Odum EP (1955). Trophic Structure and Productivity of a Windward Coral Reef Community on Eniwetok Atoll. Ecological Monographs.

[31] Moberg FF, Folke C (1999). Ecological goods and services of coral reef ecosystems. Ecol. Econ..

[32] Connell JH, Series N, Mar N (1978). Diversity in Tropical Rain Forests and Coral Reefs High diversity of trees and corals is maintained. Science (80-.)..

[33] Hughes TP (2003). Climate change, human impacts, and the resilience of coral reefs. Science.

[34] Hughes TP, Bellwood DR, Connolly SR, Cornell HV, Karlson RH (2014). Double Jeopardy and Global Extinction Risk in Corals and Reef Fishes. Curr. Biol..

[35] Bellwood DR, Hughes TP, Folke C, Nyström M (2004). Confronting the coral reef crisis. Nature.

[36] Hughes TP (2017). Global warming and recurrent mass bleaching of corals. Nature.

[37] Hoegh-Guldberg O (2007). Coral reefs under rapid climate change and ocean acidification. Science (80-.)..

[38] Lecchini D, Planes S, Galzin R (2007). The influence of habitat characteristics and conspecifics on attraction and survival of coral reef fish juveniles. J. Exp. Mar. Bio. Ecol..

[39] Russ G (1984). Distribution and abundance of herbivorous grazing fishes in the central Great Barrier Reef. I. Levels of variability across the entire continental shelf. Mar. Ecol. Prog. Ser..

[40] Hughes TP (2007). Phase Shifts, Herbivory, and the Resilience of Coral Reefs to Climate Change. Curr. Biol..

[41] Leis, J. M. & McCormick, M. I. In *Coral Reef Fishes: Dynamics and Diversity in a Complex Ecosystem* 171–199 (2002).

[42] Planes S, Lecaillon G (2001). Caging experiment to examine mortality during metamorphosis of coral reef fish larvae. Coral Reefs.

[43] McCormick MI, Makey LJ, Dufour V (2002). Comparative study of metamorphosis in tropical reef fishes. Mar. Biol..

[44] Doherty PJ (2004). High mortality during settlement is a population bottleneck for a tropical surgeonfish. Ecology.

[45] Lecchini D, Shima JS, Banaigs B, Galzin R (2005). Larval sensory abilities and mechanisms of habitat selection of a coral reef fish during settlement. Oecologia.

[46] Lecchini D, Planes S, Galzin R (2005). Experimental assessment of sensory modalities of coral-reef fish larvae in the recognition of their settlement habitat. Behav. Ecol. Sociobiol..

[47] Almany GR, Webster MS (2006). The predation gauntlet: early post-settlement mortality in reef fishes. Coral Reefs.

[48] Frédérich B, Colleye O, Lepoint G, Lecchini D (2012). Mismatch between shape changes and ecological shifts during the post-settlement growth of the surgeonfish, *Acanthurus triostegus*. Front. Zool..

[49] Lecchini D, Nakamura Y (2013). Use of chemical cues by coral reef animal larvae for habitat selection. Aquat. Biol..

[50] Doherty, P. J. In Coral Reef Fishes: Dynamics and Diversity in a Complex Ecosystem 327–355 (2002).

[51] Dixson DL, Abrego D, Hay ME (2014). Chemically mediated behavior of recruiting corals and fishes: A tipping point that may limit reef recovery. Science (80-.)..

[52] Huijbers CM (2012). A test of the senses: fish select novel habitats by responding to multiple cues. Ecology.

[53] O’Connor JJ (2016). Sediment pollution impacts sensory ability and performance of settling coral-reef fish. Oecologia.

[54] Barth P (2015). From the ocean to a reef habitat: how do the larvae of coral reef fishes find their way home? A state of art on the latest advances. Life Environ..

[55] Ferrari MCO (2015). The effects of background risk on behavioural lateralization in a coral reef fish. Funct. Ecol..

[56] Ferrari MCO (2015). Living in a risky world: the onset and ontogeny of an integrated antipredator phenotype in a coral reef fish. Sci. Rep..

[57] Nilsson GE (2012). Near-future carbon dioxide levels alter fish behaviour by interfering with neurotransmitter function. Nat. Clim. Chang..

[58] Domenici P, Allan BJM, Watson S-A, McCormick MI, Munday PL (2014). Shifting from right to left: the combined effect of elevated CO2 and temperature on behavioural lateralization in a coral reef fish. PLoS One.

[59] Domenici P, Allan B, McCormick MI, Munday PL (2012). Elevated carbon dioxide affects behavioural lateralization in a coral reef fish. Biol. Lett..

[60] Carvalho FP (2002). Distribution, fate and effects of pesticide residues in tropical coastal lagoons of northwestern Mexico. Environ. Technol..

[61] Islam MS, Tanaka M (2004). Impacts of pollution on coastal and marine ecosystems including coastal and marine fisheries and approach for management: A review and synthesis. Mar. Pollut. Bull..

[62] Fabricius KE (2005). Effects of terrestrial runoff on the ecology of corals and coral reefs: Review and synthesis. Mar. Pollut. Bull..

[63] Laabs V, Wehrhan A, Pinto A, Dores E, Amelung W (2007). Pesticide fate in tropical wetlands of Brazil: An aquatic microcosm study under semi-field conditions. Chemosphere.

[64] Leong KH, Benjamin Tan LL, Mustafa AM (2007). Contamination levels of selected organochlorine and organophosphate pesticides in the Selangor River, Malaysia between 2002 and 2003. Chemosphere.

[65] Lewis SE (2009). Herbicides: A new threat to the Great Barrier Reef. Environ. Pollut.

[66] Packett R, Dougall C, Rohde K, Noble R (2009). Agricultural lands are hot-spots for annual runoff polluting the southern Great Barrier Reef lagoon. Mar. Pollut. Bull..

[67] Shaw M (2010). Monitoring pesticides in the Great Barrier Reef. Mar. Pollut. Bull..

[68] Roche H, Salvat B, Ramade F (2011). Assessment of the pesticides pollution of coral reefs communities from French Polynesia. Rev. d’écologie (Terre vie).

[69] Kroon FJ (2012). River loads of suspended solids, nitrogen, phosphorus and herbicides delivered to the Great Barrier Reef lagoon. Mar. Pollut. Bull..

[70] Cavanagh JE, Burns KA, Brunskill GJ (1999). & Coventry, R. J. Organochlorine pesticide residues in soils and sediments of the Herbert and Burdekin River Regions, North Queensland - Implications for contamination of the Great Barrier Reef. Mar. Pollut. Bull..

[71] Botté ES, Jerry DR, Codi King S, Smith-Keune C, Negri AP (2012). Effects of chlorpyrifos on cholinesterase activity and stress markers in the tropical reef fish Acanthochromis polyacanthus. Mar. Pollut. Bull..

[72] Deb N, Das S (2013). Chlorpyrifos Toxicity in Fish: A Review. Curr. World Environ..

[73] Kavitha P, Rao JV (2008). Toxic effects of chlorpyrifos on antioxidant enzymes and target enzyme acetylcholinesterase interaction in mosquito fish, Gambusia affinis. Environ. Toxicol. Pharmacol..

[74] Slotkin TA, Cooper EM, Stapleton HM, Seidler FJ (2013). Does thyroid disruption contribute to the developmental neurotoxicity of chlorpyrifos?. Environ. Toxicol. Pharmacol..

[75] Salvat B, Roche H, Berny P, Ramade F (2012). Recherches sur la contamination par les pesticides d’organismes marins des réseaux trophiques récifaux de Polynésie Française. Rev. d’écologie.

[76] Randall JE (1961). A contribution to the biology of the convict surgeonfish of the Hawaiian Islands. Acanthurus triostegus sandvicensis. Pacific Sci..

[77] Sovrano VA, Andrew RJ (2006). Eye use during viewing a reflection: Behavioural lateralisation in zebrafish larvae. Behav. Brain Res..

[78] NRA. in National Registration Authority for Agricultural and veterinary Chemicals **1**, 17 (2000). 11301731

[79] Sovrano VA, Bertolucci C, Frigato E, Foà A, Rogers LJ (2016). Influence of exposure in ovo to different light wavelengths on the lateralization of social response in zebrafish larvae. Physiol. Behav..

[80] Vallortigara G, Andrew RJ (1994). Differential involvement of right and left hemisphere in individual recognition in the domestic chick. Behav. Processes.

[81] Zucca P, Sovrano VA (2008). Animal lateralization and social recognition: Quails use their left visual hemifield when approaching a companion and their right visual hemifield when approaching a stranger. Cortex.

[82] Lecchini D, Osenberg CW, St Shima JS, Mary CM, Galzin R (2007). Ontogenetic changes in habitat selection during settlement in a coral reef fish: ecological determinants and sensory mechanisms. Coral Reefs.

[83] Bibost AL, Brown C (2014). Laterality influences cognitive performance in rainbowfish Melanotaenia duboulayi. Anim. Cogn..

[84] Miklosi A, Andrew RJ, Savage H (1997). Behavioural lateralisation of the tetrapod type in the zebrafish (Brachydanio rerio). Physiol. Behav..

[85] Cantalupo C, Bisazza A, Vallortigara G (1995). Lateralization of predator-evasion response in a teleost fish (Girardinus falcatus). Neuropsychologia.

[86] De Santi A, Sovrano VA, Bisazza A, Vallortigara G (2001). Mosquitofish display differential left- and right-eye use during mirror image scrutiny and predator inspection responses. Anim. Behav..

[87] Bigot M (2016). Air-Seawater Exchange of Organochlorine Pesticides in the Southern Ocean between Australia and Antarctica. Environ. Sci. Technol..

[88] Fulton MH, Key PB (2001). Acetylcholinesterase inhibition in estuarine fish and invertebrates as an indicator of organophosphorus insecticide exposure and effects. Environ. Toxicol. Chem..

[89] Ledlie MH (2007). Phase shifts and the role of herbivory in the resilience of coral reefs. Coral Reefs.

[90] Dufour V, Galzin R (1993). Colonization patterns of reef fish larvae to the lagoon at Moorea Island, French Polynesia. Mar. Ecol. Prog. Ser..

[91] Lecchini D, Dufour V, Carleton JH, Strand S, Galzin R (2004). Estimating the patch size of larval fishes during colonization on coral reefs. J. Fish Biol..

[92] Lo-Yat A (2011). Extreme climatic events reduce ocean productivity and larval supply in a tropical reef ecosystem. Glob. Chang. Biol..

[93] Dufour V, Riclet E, Lo-Yat A (1996). Colonization of reef fishes at Moorea island, french polynesia: temporal and spatial variation of the larval flux. Mar. Freshw. Res..

[94] Besson M (2017). Consistency in the supply of larval fishes among coral reefs in French Polynesia. PLoS One.

[95] McCormick MI (1999). Delayed metamorphosis of a tropical reef fish (*Acanthurus triostegus*): a field experiment. Mar. Ecol. Prog. Ser..

